# Relationship Between Work Engagement, Psychosocial Risks, and Mental Health Among Spanish Nurses: A Cross-Sectional Study

**DOI:** 10.3389/fpubh.2020.627472

**Published:** 2021-01-26

**Authors:** Juan Jesús García-Iglesias, Juan Gómez-Salgado, Mónica Ortega-Moreno, Yolanda Navarro-Abal

**Affiliations:** ^1^Department of Sociology, Social Work and Public Health, Faculty of Labour Sciences, University of Huelva, Huelva, Spain; ^2^Nursing Department, Atlântica Health School, Barcarena, Portugal; ^3^Safety and Health Postgraduate Programme, Universidad Espíritu Santo, Guayaquil, Ecuador; ^4^Department of Economy, Faculty of Economic and Business Sciences, University of Huelva, Huelva, Spain; ^5^Department of Social, Evolutionary and Educational Psychology, Faculty of Labour Sciences, University of Huelva, Huelva, Spain

**Keywords:** health personnel, mental health, work engagement, occupational disease, nurses, primary health care, emergency medical services

## Abstract

**Background:** Exposure to risk factors may lead to health problems of varied nature and to an increased risk of suffering accidents at work.

**Objectives:** The aim of this study was to evaluate the work engagement, psychosocial risks, and psychological well-being of Spanish nurses, analyzing existing relationships, and their associations with self-reported mental health problems of nurses.

**Methods:** To this end, a cross-sectional observational study was carried out with a sample of 1,704 Spanish nurses between January 2019 and January 2020, using a self-administered questionnaire containing sociodemographic variables, the Spanish version of the Copenhagen Psychosocial Questionnaire (CoPsoQ-istas21), the Utrecht Work Engagement Scale (UWES-9), and the General Health Questionnaire (GHQ-12).

**Results:** The Kruskal-Wallis test showed that nurses' perceptions for each of the tests significantly differed among different healthcare areas (*p* < 0.05). The results indicated that emergency nurses offered higher scores in all dimensions of the CoPsoQ-istas21 and GHQ-12 tests; and in primary care, nurses scored higher in all three dimensions of the UWES-9 test. In addition, self-perceived health and vigor at work were identified as predictive factors of mental health.

**Conclusions:** A high percentage of Spanish nurses perceived a high level of psychosocial risk in the exercise of their duties and nearly 41% could suffer from some mental health-related problem. Primary Care nurses showed higher levels of work engagement and lower perception of psychosocial risks than Emergency nurses. Results may allow to identify a professional profile which is more likely to suffer from psychological distress, as both the working conditions and the work commitment expressed by nurses in their daily work are key elements in assessing the possible psychosocial risks to which they may be exposed.

## Introduction

The health sector is considered to be one of the most exposed to occupational risks (1). The health worker may have his health compromised by frequent, continuous, and persistent exposure to physical, chemical, biological, ergonomic, and psychosocial risks of various kinds and nature (2). These idiosyncratic working conditions related, for example, to intensive and rotating work shifts, high levels of stress and high psychological demands, among others, may affect the development of psychological disorders, as is the case of nursing professionals (3).

In this context, perspectives are required to refocus understanding and intervention in the psychosocial context and its interaction with the person, focusing on premises that value the positive capital of people and the organizational context in which they develop occupationally and personally. Relevance should focus not only on the risk factors that can have negative consequences, but on the protective factors that can facilitate the prevention of such risks, leading to subjective welfare states by the healthcare professional (4). Within this perspective, importance has been given to the study of engagement as a shield against burnout, that has its own entity (5). Engagement is understood as an emotional, cognitive, and psychological construct that refers to a positive and satisfactory mental state related to work, based on three dimensions: vigor (high energy levels and mental endurance), dedication (work involvement, enthusiasm, and challenge by work), and absorption (total concentration at work) (6).

In this sense, having committed employees increases productivity levels, results in greater enthusiasm and interaction with colleagues, more motivation and creativity, and a decrease in absenteeism levels and the number of errors (5). To do this, it is necessary for personal factors such as situational factors to encourage increased levels of engagement.

In recent decades, from this approach of positive psychology, special emphasis has been placed on the relationship between health, well-being, and happiness, being sufficiently and scientifically proven at this point that the evidence of these variables in the person brings about consequences that are considered desirable, at the very least, and in the working context (7, 8).

According to the results of the 6th European Working Conditions Survey (9), nurses, due to their working conditions, present a clear and remarkable increase in reporting mental health problems and psychological discomfort (7, 8), which mainly manifest in irritability, stomach pain, sleep problems, and anxiety-like symptoms (9). In this sense, physical health is also affected, and a greater number of symptoms are observed, as compared to the rest of the working population.

Nurses experience recurrent symptomatology related to unfavorable psychosocial conditions as a result of their work performance: high levels of stress, anxiety, emotional overload, or fatigue, mainly as a result of the nature of their work and the place where they perform their work (10, 11). These adverse psychosocial circumstances are also related to reduced quality of life in their self-perceived health levels and also regarding well-being (5). Similarly, there is an increase in absenteeism and the number of cardiovascular, musculoskeletal, and mental health-related diseases. These effects, according to the Job Demands-Resources Model by Bakker et al. (12) may be explained by the imbalance between resources and demands, where work demands are more numerous than work and/or personal resources.

Adverse working conditions affecting the health of nurses have been identified in very different work environments. Primary care (PC) nurses are subjected to a shortage of time to perform their duties and this fact may be related to their patient quota, excessive bureaucratic tasks (11), being subjected to geographical dispersion by having to assist outside their health centre and, in many cases, having to treat a population with a high level of dependence and complexity (13). In the case of intensive care units (ICU) and hospital emergency services (HES), nurses may be exposed to stressful situations due to the critical situation of the patient, and especially in HES (14, 15), where unpredictable events must be dealt with and being this the service where nurses are most exposed to aggression (16). As has been observed in nurses working in emergency and out-of-hospital emergency services (OHES), a high presence of symptomatology related to work stress (physical and emotional fatigue, overload, tension, and anxiety) has been observed that may pose a risk of impaired mental health to nurses working in these environments (17, 18).

As a result of this evidence, over the last few years, concern for the occupational health of the nursing professional collective has led to the development of studies related to pathologies and traits linked to professional performance (19). The data obtained show the need to address this problem that causes suffering and professional and personal impairment, and which directly affects the quality of the care provided (5).

As mentioned, there is evidence that the variability of the work environment of the nursing profession conditions the risk factors to which professionals are exposed. This study raises the hypothesis that the profile of the work environment influences the psychosocial risk endured by nurses and its effect on their mental well-being. It would be interesting to compare the psychosocial impact of different clinical settings to identify professionals at greatest risk and design preventive interventions adapted to each reality. The objective of this study was to describe the work engagement, psychosocial risks, and psychological well-being of a sample of Spanish nurses belonging to different areas of care, analyzing the relationships between these variables and their associations with self-reported mental health problems of nurses.

## Materials and Methods

### Study Design and Participants

A cross-sectional study was conducted on a population of 185,835 Spanish nurses. According to the National Institute of Health Management (*INGESA*, for its acronym in Spanish) (20) under the Ministry of Health, in 2018 there were 185,835 nurses working in the National Health System, of which 30,499 worked in PC, 150,269 in specialized care (8,101 were part of the HES), 3,061 in the OHES and 15,716 nurses worked in the private health sector (of which 1,754 worked in private HES).

For this population, a necessary sample of 386 nurses was estimated for a 5% error, with a 95% confidence and a heterogeneity of 50%. As inclusion criteria, the following were established: (i) nurses who carry out healthcare, teaching, management and/or research work typical of their profession; (ii) resident in the national territory (Spain); (iii) active nurses; and (iv) who have accepted the informed consent. The exclusion criteria were: (i) not performing strictly nursing tasks; (ii) Spanish nurses working outside Spain at present.

An accidental or causal sampling method was followed, obtaining a sample of 1,808 participants. Of the total, those questionnaires that were not fully completed or had inconsistencies were eliminated, so 5.75% of them were removed from the study (losses), leaving a final sample of 1,704 participants.

Finally, participants were classified in three groups according to where they performed their duties: A group of PC nurses, a group of EC nurses (emergency care nurses of HES-OHES), and a group that included the rest of nurses. These were the arranged groups, as they are considered the most heterogeneous in terms of functions, stress levels, types of shifts and working hours, severity of the patient to assist, and professional profile (13, 21–23).

### Instruments

The questionnaire distinguishes four parts: (1) sociodemographic variables; (2) psychosocial risk assessment; (3) engagement assessment; and (4) psychological well-being assessment and detection of non-psychotic psychiatric problems.

Basic sociodemographic information included sex, age, place of residence, type of entity where they worked, type of position they occupied, type of service, time working at the current centre, type of employment contract, working hours (type of work, working days per week, out-of-hour tasks, shift changes), financial benefit they received, absences from work (work leave), and substance use.

The Spanish version of the Copenhagen Psychosocial Questionnaire (CoPsoQ-istas21) (24) was used for the assessment of psychosocial risks. CoPsoQ-istas21 is an evaluation tool aimed at assessing psychosocial risks, identifying and locating such risks and facilitating the design and implementation of preventive measures. This instrument consists of 5 dimensions that are: Psychological Requirements, Active Work and Skills Development, Social Support in the Company, Compensation, and Double Presence. In relation to reliability, it has an internal consistency with a high Cronbach alpha (α = 0.92). The simple summation of the total points made it possible to determine the score for each dimension. This score calculated the number of workers at low, medium, or high-risk levels. Low (L), medium (M), and high (H) levels had different scores depending on the dimension: Psychological demands (L: 0–8; M: 9–11; H: 12–20), Active work and skills development (L: 0–5; M: 6–8; H: 9–20), Social support in the company (L: 0–3; M: 4–6: H: 7–20); Compensation (L: 0–2; M: 3–5; H: 6–12), and Double Presence (L: 0–1; M: 2–3; H: 4–8).

The Utrecht Work Engagement Scale (UWES) (25) was the instrument used to assess engagement at work and it consists of nine items distributed in three items for each dimension (vigor, dedication, and absorption), with a Likert scale of seven points which ranges from “never or not once” to “always or every day.” Cronbach's alpha reliability indexes are as follows: vigor (α = 0.82), dedication (α = 0.86), and absorption (α = 0.8).

The UWES survey gave three partial mean scores, corresponding to each subscale, and a total score within the range of 0–6 points. In addition, percentages of the score were compared according to the following recoding: 1 (Sometimes per year) from 0 to 0.99; 2 (Once or less per month) from 1 to 1.99; 3 (Sometimes per month) from 2 to 2.99; 4 (Once a week) from 3 to 3.99; 5 (Sometimes per week) from 4 to 4.99; and 6 (Every day) from 5 to 6.

The General Health Questionnaire (GHQ-12) (26) is a self-administered screening test that evaluates psychological well-being and detects non-psychotic psychiatric problems. It consists of 12 items: 6 positive and 6 negative sentences. GHQ-12 has proven good reliability in the different studies carried out, with Cronbach alphas ranging from 0.76 to 0.86 in the Spanish population (27).

Responses are valued on a Likert scale from 0 to 3 points. Scores 0 and 1 were recoded as 0, and scores 2 and 3, as 1. The total score was calculated by adding the scores obtained in all items of the dichotomous scale and 3 was considered a breakpoint for this one-dimensional screening instrument, with a range between 0 and 12 points. The percentage of individuals considered to present high values in terms of impaired mental health was determined, based on the number of individuals with higher atypical scores.

### Procedure

Data were collected through an online questionnaire conducted by three psychologists and two nurses. The questionnaire was distributed by the General Council of Nursing and the Nursing Colleges of each Spanish province through a web link to Google Forms^©^, being disseminated through their official webpages and social networks. Likewise, the link was shared in social networks via WhatsApp, Twitter, and LinkedIn.

Data collection took place between January 2019 and January 2020.

### Statistical Analysis

Descriptive statistics were presented as percentages and frequencies. The Kolmogorov-Smirnov test was used to determine whether the data exhibited normal behavior. The Kruskal-Wallis test was used to check for differences in the analyzed groups with respect to the assessment of the different dimensions, and the Mann–Whitney *U-*test with Bonferroni correction was used to analyse which subgroups differed from each other.

The bivariate analysis between the variables under study and having or not psychological distress shows the value of statistical significance with Chi-squared, the estimated risks from the Odds Ratios (OR), and their confidence intervals. Finally, in order to predict the probability that a healthcare professional has to present distress, a logistic regression analysis was carried out based on those factors that, after the preliminary analytical study, were considered most influential (sex, age, service in which the professional works, perceived general health, UWES vigor, UWES dedication, UWES absorption, and total UWES).

Previous analysis of the data suggested that the working group could have a confounding effect on the variables to be included in the model. In order to identify this effect, statistical significance was assessed by chi-squared, as well as risks and confidence intervals of the stratified analysis by differentiating the data as they belonged to PC, HES-OHES, or other areas with those variables with the greatest risk, obtaining significant differences in all cases. The contrast of OR values by strata led to the conclusion that, in all independent variables under study, there were significant differences between the groups (*p* < 0.001). Therefore, to avoid confusion in the model, it was chosen to determine the most optimal model in each working group, avoiding excessive interactions within the same model.

SPSS version 20.0 software was used for the study.

### Ethical Considerations

Participants voluntarily responded to the questionnaire and accepted the informed consent. The questionnaire explained in detail the study subject matter and included the participant's consent. Participants' responses were recorded anonymously, and the information was treated confidentially.

The study was conducted under the “Ethical Principles for Medical Research Involving Humans” contained in the latest version of the Helsinki Declaration (Fortress Amendment, Brazil, October 2013). It was also approved by the Ethics Committee of the General Council of Nursing (Spain) in April 2018.

The data obtained during the study were processed in accordance with Organic Law 3/2018 of December 5 on the Protection of Personal Data and guarantee of digital rights.

## Results

### Sample Characteristics

Of the 1,704 participants who provided analysable data (loss of 5.75% compared to 1,808), 18.1% of them (308) were PC nurses, 8.7% (149) were EC nurses, and 73.2% (1,247) belonged to the other areas. The mean age of the people surveyed was 41.69 years (standard deviation 10.79), with 86.3% of them being females.

Most participants worked in a public or associated hospital (59.91%, 1,020), 18.08% in a health centre (308), and the rest in other institutions (22.01%, 375). 49.01% (801) of participants had remained in their current workplace for more than 10 years. They were civil servants 27.73% (484), permanent (indefinite contract) 28.99% (484), interim 21.03% (358), and temporary 19.44% (360). The rest of contractual relationships (discontinuous-permanent, temporary with training contract, etc.) accumulate < 1.5% of the cases each. 92.47% (1,567) of the surveyed nurses have a full-time contract, with a reduction in working hours in 9.89% of these cases. Similarly, they perform tasks that they consider to correspond to their professional category 70.64% (1,188), while 20.54% (362) think their work is above their category, and the rest, below (3.67%; 73) or do not know (5.05%; 81). The most common working hours include working both weekdays and weekends and holidays in 54.97% (968) of cases, or from Monday to Friday in 31.47% (525) of cases. The mean distance from the place of residence to the work centre was 16.68 km (standard deviation 25). 24.08% (441) of the participants earn a net amount per month ≤ 1,500 euros; ~50% (47.52%, 838) receive between 1,501 and 2,100 euros; 17.2% (263) receive up to 2,400 euros, 11.21% (162) receive more.

### Test Assessment

The number of cases, percentages, mean scores, and deviations typical of the three analyzed tests (CoPsoQ-istas21, UWES, and GHQ) for each dimension are shown in Table 1, both globally and by type of service.

**Table 1:** Descriptive results of the three variables: psychosocial risks (CoPsoQ-istas21), work engagement (UWES), and psychological well-being (GHQ).

		**PC nurses**	**EC nurses**	**Other areas**	**Overall total**	**Independent sampling Kruskal-Wallis test**	
	Number of cases	308	149	1,247	1,704		
	Percentage no of cases	18.08%	8.74%	73.18%	100%		
		**Mean (SD)**	**Mean (SD)**	**Mean (SD)**	**Mean (SD)**	**χ^2^**	***p***
**CoPsoQ-istas21**	Psychological Requirements (0–20)	11.97 (2.80)	13.96 (2.75)	13.54 (2.82)	13.29 (2.88)	82.460	0.000
	Active Work and Skills Development (0–20)	7.75 (3.22)	9.47 (3.05)	8.65 (3.16)	8.56 (3.19)	33.261	0.000
	Social Support for the Company (0–20)	7.60 (3.37)	9.03 (3.20)	8.44 (3.19)	8.34 (3.24)	24.004	0.000
	Compensation (0–12)	4.60 (2.77)	5.50 (2.64)	5.29 (2.82)	5.19 (2.81)	19.832	0.000
	Double Presence (0–8)	3.37 (1.64)	3.81 (1.74)	3.52 (1.68)	3.52 (1.68)	7.464	0.024
**UWES**	Vigor (0–6)	4.44 (1.29)	4.00 (1.35)	3.98 (1.36)	4.06 (1.36)	34.029	0.000
	Dedication (0–6)	4.70 (1.32)	4.39 (1.49)	4.31 (1.45)	4.39 (1.44)	21.777	0.000
	Absorption (0–6)	4.63 (1.28)	4.07 (1.56)	4.21 (1.42)	4.27 (1.42)	21.150	0.000
	UWES_Total (0–6)	4.59 (1.21)	4.15 (1.38)	4.17 (1.30)	4.24 (1.30)	31.886	0.000
**GHQ**	General Health Questionnaire	2.94 (3.41)	4.17 (3.79)	3.87 (3.70)	3.73 (3.67)	19.778	0.000

*PC, Primary Care; EC, Emergency Care; SD, Standard deviation*.

Mann–Whitney *U-*test for independent samples with Bonferroni correction showed that perceptions of nurses significantly differed depending on the service. The statistics and *p-*values associated with this test were listed in Table 2.

**Table 2:** Comparison between psychosocial risks (CoPsoQ-istas21), work engagement (UWES), and psychological well-being (GHQ) according to the type of nursing service.

**Mann–Whitney** ***U-*****test for two independent samples**		**PC nurses** ***vs*****. EC nurses**		**PC nurses** ***vs*****. Other areas**		**EC nurses** ***vs***. **Other areas**	
**Test**	**Dimension**	**Statistical**	***p***	**Statistical**	***p***	**Statistical**	***p***
**CoPsoQ-istas21**	Psychological Requirements (0–20)	14006.00^**^	0.000	252658.00^**^	0.000	85641.00	0.116
	Active Work and Skills Development (0–20)	15871.50^**^	0.000	223618.00^**^	0.000	79275.50^*^	0.003
	Social Support for the Company (0–20)	17170.50^**^	0.000	221095.00^**^	0.000	83682.50^*^	0.046
	Compensation (0–12)	18253.50^**^	0.000	221130.50^**^	0.000	88538.50	0.345
	Double Presence (0–8)	19425.50^*^	0.007	203391.50	0.101	84151.50	0.055
**UWES-9**	Vigor (0–6)	27469.00^**^	0.001	151159.50^**^	0.000	91903.00	0.829
	Dedication (0–6)	25636.50^*^	0.040	159217.50^**^	0.000	88572.50	0.350
	Absorption (0–6)	27744.00^**^	0.000	156665.00^**^	0.000	96429.00	0.446
	UWES_Total (0–6)	27297.50^**^	0.001	152308.00^**^	0.000	92034.00	0.852
**GHQ-12**	General Health Questionnaire –Likert scale	18307.00^**^	0.000	219137.50^**^	0.000	88031.50	0.294
	General Health Questionnaire –Dichotomous scale	18290.00^**^	0.000	220838.50^**^	0.000	88174.00	0.305

* *p < 0.05*,

** *p < 0.001*.

*PC, Primary Care; EC, Emergency Care*.

### Work Engagement Level

More than 75% of PC nurses achieved a mean percentage of 5 or 6 in all subscales and in the total score. For EC nurses, the percentages associated with means of 5 or 6 were 57.72% regarding vigor, 71.14% for dedication, and 62.42% in absorption. However, for the group in the rest of the areas, the percentages were 58.46, 69.61, and 69.81%, respectively, and in both services, this percentage of the total was slightly higher than 62%. Specifically, in all areas under study, more than 30% reported experiencing these feelings every day, as compared to less than a mean value just over 2% who does sometimes per year (Supplementary Material 1).

### Psychosocial Risk Assessment

**Figure 1:**
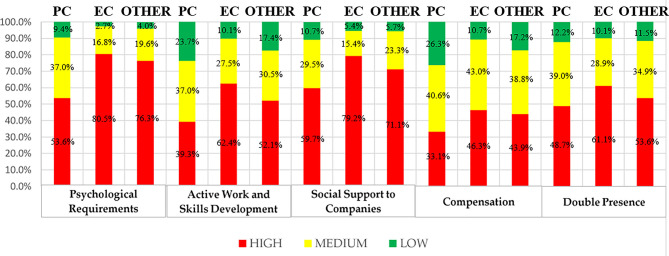
Risk levels by dimension and service (CoPsoQ-istas21).

Most study participants had a high psychosocial risk, as more than 50% of them perceive a high level of risk (red color), except in the Compensation dimension, in which they showed a medium-high level (Figure 1).

### Psychological Well-Being Assessment

The assessment of the GHQ test stands out for a central positioning of the values, as well as the asymmetry on the right in all cases. 41.14% (701) of professionals scored with values > 3 in the GHQ test. This percentage was lower for PC nurses (30.52%, 94), slightly higher for EC nurses (47.65%, 71), and very similar in other areas (42.98%, 536) (Supplementary Material 2).

### Binary Logistic Regression

The logistic regression analysis was performed on the basis of factors that were considered most influential after the preliminary study. These independent variables were: sex, age, working group (PC, EC, or other areas), perceived general health, score in vigor, dedication, absorption scales, and total in the engagement questionnaire (UWES).

Bivariate analysis, between study variables and having or not distress, shows the Chi-squared value of statistical significance, estimated risks from Odds Ratio (OR), and their confidence intervals (Table 3).

**Table 3:** Bivariate analysis between study variables and having or not psychological distress^*^.

	**TOTAL**	**GHQ≤3**	**GHQ>3**	**χ^2^ (*p*)**	**OR (95% CI)**
	**N (%)**	**N (%)**	**N (%)**		
**Total**	1,704 (100)	1,003 (58.86)	701 (41.14)		
**Sex**					
0. Male	234	158 (67.5)	76 (32.5)		
1. Female	1,470	845 (57.5)	625 (42.5)	8.401 (0.004)	1.538 (1.148, 2.060)
Age^**^					
0. Older than 41	833	541 (64.9)	292 (35.1)	26.723 (< 0.001)	1.676 (1.377, 2.040)
1.41 or younger	840	441 (52.5)	399 (47.5)		
**Service group**					
0. PC Nurses	308 (8.7)	214 (69.5)	94 (30.5)		
1. EC Nurses	149 (18.1)	78 (52.3)	71 (47.7)	12.776 (< 0.001)	2.072 (1.385, 3.100)
2. Other areas	1,247 (73.2)	711 (57.0)	536 (43.0)	15.921 (< 0.001)	1.716 (1.314, 2.242)
**General Perceived Health**					
0. Optimal	1,457	927 (63.6)	530 (36.4)		
1. Mediocre or poor	247	76 (30.08)	171 (69.2)	94.146 (< 0.001)	3.935 (2.943, 5.262)
**UWES Vigor**					
0. At least once a week	1,373	903 (65.8)	470 (34.2)		
1. Less than once a week	331	100 (30.2)	231 (69.8)	139.251 (< 0.001)	4.428 (3.423, 5.755)
**UWES Dedication**					
0. At least once a week	1,447	925 (63.9)	522 (36.1)		
1. Less than once a week	257	78 (30.4)	179 (69.6)	101.599 (< 0.001)	4.067 (3.053, 5.417)
**UWES Absorption**					
0. At least once a week	1,439	908 (63.1)	531 (36.9)		
1. Less than once a week	265	95 (35.8)	170 (64.2)	68.628 (< 0.001)	3.060 (2.328, 4.020)
**UWES Total**					
0. At least once a week	1,398	904 (64.7)	494 (35.3)		
1. Less than once a week	306	99 (32.4)	207 (67.6)	108.237 (< 0.001)	3.826 (2.940, 4.979)

* Numbering before the modalities of each variable indicates the encoding method used for the analysis, coinciding value “0” with the baseline or reference category, and values “1” or “2” with categories that the researcher considers risky;

** *total cases per variable do not correspond to the total number of professionals because data was not collected for some professionals; OR, Odds Ratio; 95% CI, Confidence Interval level at the 95%; PC, Primary Care; EC, Emergency Care; OR, Odds Ratio; χ^2^, Chi-squared*.

All the bivariate contrasts made were significant, which indicated the relationship between the variables, positive in the case of OR values greater than the unit. It is worth noting that health professionals with a mediocre or poor perception of health had a 3.935 ([2.943; 5.262]) higher risk of suffering distress than professionals with an optimal perception of overall health.

Prior to the construction of the models, a new bivariate analysis was performed between the variables under study and whether or not having distress, in each of the three working groups. Table 4 lists the significant variables in each of the binary regression models, the estimated risks from the Odd Ratios (OR), and confidence intervals for these models. In the three models, PC nurses, EC nurses, and those from other areas, the perceived health and subscale variables of the UWES test were predictive. Age was significant in the groups of PC and of other nursing areas, while sex and the dedication subscale of the UWES test were only significant in the last group. The absorption subscale and the total scale of the UWES test were not significant in any of the three models, so they were not shown in the table. The Hosmer and Lemeshow test showed no statistical significance (lp > 0.005 in all three models), which indicated a good fit in the logistic regression model. On the other hand, the omnibus test made it possible to state that the variables included in the model, taken together, help explain the modifications that occur in the likelihood of having psychological distress (*p* < 0.001).

**Table 4:** Binary logistic regression model for psychological distress by specialty.

**Variables**		**PC Nurses**	**EC Nurses**	**Other areas**
		**OR (95% CI)**	**OR (95% CI)**	**OR (95% CI)**
Sex (ref. Male)		NA	NA	1.682^*^ (1.157, 2.444)
Age (ref. Older than 41)		1.924^*^ (1.092, 3.390)	NA	1.612^**^ (1.257, 2.067)
Perceived Health (ref. Optimal)		4.448^**^ (2.134, 9.273)	3.040^*^ (1.080, 8.546)	3.382^**^ (2.340, 4.890)
UWES (ref. At least once a week)	Vigor-UWES	3.052^*^ (1.308, 7.121)	3.625^*^ (1.520, 8.641)	2.561^**^ (1.786, 3.673)
	Dedication-UWES	NA	NA	2.031^**^ (1.354, 3.047)
Sensitivity/Specificity		90.5%/40.4%	82.9%/48.6%	84.2%/47.2%
Correctly classified percentage		75.1%	66.4%	68.2%
R^2^		0.186	0.145	0.174
Hosmer-Lemoshov test		χ^2^ = 0.630 (*p* = 0.730)	χ^2^ = 1.837 (*p* = 0.399)	χ^2^ = 4.342 (*p* = 0.501)
Omnibus test		χ^2^ = 43.135 (*p* < 0.001)	χ^2^ = 16.744 (*p* < 0.001)	χ^2^ = 169.485 (*p* < 0.001)

* *p < 0.05*,

** *p < 0.001. NA, variables that are not present in the model; OR, Odds Ratio; 95% CI, Confidence Interval at the 95% level*.

*PC, Primary Care; EC, Emergency Care; CI, Confidence Interval; OR, Odds Ratio; χ2, Chi-squared*.

In the proposed model for PC nurses, predictive capacity was 18.6%, correctly classifying 75.1% of professionals, with a sensitivity (proportion of professionals without distress correctly classified) of 90.5%, and a specificity (proportion of professionals without distress correctly classified) of 40.40%. PC nurses with a mediocre or poor perception of health had 4,448 (95% CI = [2.134; 9.273]) times higher risk of psychological distress than those professionals with an optimal health perception. For those where vigor is not present at least once a week, the risk was 3.052 (95% CI = [2.308; 7.121]) times higher and it was also higher in professionals 41 years of age or younger (OR = 1.924; 95% CI = [1.092; 3.390]).

In the model presented for EC nurses, the modalities that had the highest weight were having a mediocre or poor perception of health (OR = 3.040; 95% CI = [1.080; 8.546]) and vigor less than once a week (OR = 3.625; 95% CI = [1.520; 8.641]). The model correctly ranks 66.4% of EC nurses, with a sensitivity of 82.9% and a specificity of 48.6%, being 14.5% the predictive capacity.

Finally, focusing on the rest of the areas, being female (OR = 1.682; 95% CI = [1.157; 2.444]), 41 years of age or younger (OR = 1.612; 95% CI = [1.257; 2.067]), having a mediocre or poor perception of health (OR = 3.382; 95% CI = [2.340; 4.890]), and vigor and dedication less than once a week (2.561; 95% CI = [1.786; 3.673]) are the modalities with the highest risk of psychological distress. The model correctly ranks 68.2% of professionals, with a sensitivity of 84.2%, a specificity of 47.2%, and a predictive capacity of 17.4%.

## Discussion

This study found, in a sample of 1,704 Spanish nurses, how psychological risks, level of engagement, or psychological well-being presented significant differences between the different types of services, i.e., emergency services, primary care, and other areas. The results indicated that emergency nurses showed high levels of psychological risk and psychological distress, and in primary care, nurses scored higher in all three dimensions of work engagement. In addition, self-perceived health and vigor at work were identified as predictive factors of mental health.

With regard to the assessment of psychosocial risks, obtained through the CoPsoQ-istas21 questionnaire, in four of the five dimensions (Psychological Requirements, Active Work and Skills Development, Social Support for companies, and Double Presence), a high-level prevalence predominates in the three groups under study, ranging from 80.5 to 48.7%. With regard to the Compensation dimension, case percentages are higher at high levels for the groups of EC nurses and of other areas, with a predominantly intermediate level among PC nurses (40.6%). This is consistent with previous studies (14) such as that conducted on 42 Resident Internal Physicians of the San Cecilio University Hospital (Granada, Spain), where 90% of doctors perceived a high risk in the Psychological Requirements dimension, low levels for the Social Support to Companies dimension, and an intermediate risk perception for 78% of the sample, as happened in the study by González-Cabrera et al. (28) on emergency practicians in Granada (Spain). In a larger sample (29), consisting of 844 health team workers from 23 public hospitals in Cordoba, Argentina, unfavorable assessments predominated in the Psychological Requirements (57.7%), Social Support and Leadership Quality (56.2%), and Double Presence (64%) dimensions, so data from both studies are in line with those found in the present one. It should be noted in the latter study (29) the variability between the different professionals since, in the compensation dimension, high risk was more common among nurses, unlike medical staff, where the Psychological Requirements (*p* < 0.001) and Social Support and Leadership Quality dimension presented a higher frequency at the level of risk, as compared to other professionals. Social support may be a key element to minimize the negative consequences of stress, as this may be one of the most important emerging risks regarding occupational health and management (30). It is particularly striking among EC nurses that the Double Presence dimension is the fourth with the highest percentage of risk, unlike other studies (3, 29), although regarding percentages, both results are in line with percentages close to 60–65%. In contrast, the PC nurses group had a lower percentage of high risk than those from other services, and the mean risk perception was lower than 50% in all dimensions.

Engagement levels were high in a considerable number of healthcare professionals under study, exceeding mean scores above 4 out of 6 in all three dimensions. More than 30% of respondents claimed to have experienced feelings of engagement every day, as compared to < 2% who reported these feelings once a year or less. The total results showed similarities with some previous studies on nurses working in Spanish public hospitals (31), and very close to the work engagement levels found by Schaufeli and Bakker (32) in 2004 in their study on Belgian and Dutch professionals of different fields, with a mean total score of 4.3. In a study on 980 nurses from eight hospitals in Saudi Arabia (20), despite having a total UWES score of 4.1, it was observed that, in the dedication sub-dimension, a score had been obtained fairly higher than in the other two (dedication: 4.6; absorption: 3.9; vigor: 4), being observed in the studies by Aboshaiqah et al. (21), Othman et al. (33), and Wan et al. (34), and in the present study sample. People who feel dedicated to their work have a strong emotional and work involvement with their work, considering difficulties as personal challenges that can be encountered in their day-to-day work. This phenomenon could be explained by the long history that women have had in this profession, assuming the role of caregiver (21). Internationally, it has been observed, in a study (35) conducted in the city of Sao José do Rio Preto (São Paulo, Brazil) on 75 PC nurses, that vigor levels were slightly above the dedication levels, 5.2 vs. 5.3, respectively. At the national level, something similar happens with the study (31) carried out in the northeast of Spain on a sample of 373 nurses from the hospital field, where higher mean scores were observed for the vigor subscale 4.68 (SD = 1.07) than dedication 4.61 (SD = 1.37) and absorption scores 4.34 (SD = 1.24). The literature notes that levels of commitment (in their different dimensions) are influenced by sex, work service, educational level, and type of occupation (31, 34). As other authors indicate, the type of service appears to influence the three dimensions of the UWES scale. It has been observed how more than 75% of PC nurses scored high across all subscales and in the total score. Regarding the EC nurses and the group that includes the rest of professionals, these percentages are below those found in the PC group. This was the case in the study by Medeiros-Maio et al. (36), on Portuguese PC nurses who carried out their care work in Las Azores (Portugal), and in the studies by Loureno et al. (37) and da Silva et al. (35) on PC healthcare professionals in the state of São Paulo (Brazil). While true, it is striking that a considerable number of healthcare professionals scored high in the Engagement dimension, while few reported feelings of engagement, as was the case in the study by Aboshaiqah et al. (21). It is noted that, especially age (21), the characteristics of the work itself, and the environment can be predictors of work engagement (34, 35).

According to data from the present study, it is observed that four out of ten health professionals may have their mental health impaired (GHQ > 3). These figures are above those found in other studies carried out among healthcare professionals in Spain. For example, in the study by Portero et al. (38) conducted on 235 HES practicians and nurses from four Andalusian hospitals (southern Spain), 32.3% of professionals were found to have impaired mental health. Likewise, in another study carried out in a tertiary hospital in Madrid (central Spain), the differences are even more pronounced (39). In the present study, means of 3.73 (SD = 3.67) are found for the total sample in the GHQ-12, and it is striking that similar scores (3.96 [SD = 3.27]) were found in a study conducted on Japanese nurses following an earthquake in Japan in 2017 (40), unlike other studies such as that conducted by Sánchez-López et al. (41), which compares mental health between female and male nurses obtaining mean scores of 2.28 (SD = 2.81) and 1.61 (SD = 2.45), respectively. As in the general Spanish population, women refer to some mental health problems more often than men, 14.1 vs. 7.2%, figures below those found in the present study (42). This fact shows that Spanish nurses may have an increased risk of mental health problems, as compared to the general Spanish population (39). In addition, there are variables that can increase this risk such as being a woman, young (31), having a mediocre or poor perception of health, having low work commitment (34), and working in services with a high level of stress, as is the case of emergency services. By services, there have been differences of more than one point in the GHQ-12 among nurses working in PC = 2.94 (SD = 3.41), as compared to those working in emergency services 4.17 (SD = 3.79). This could be seen in the study by Abbaspour et al. (43), where 39.7% of health professionals working as OHES in Iran had high levels of risk for mental health impairment. This phenomenon can be explained by the care overload to which EC nurses are exposed. In fact, in the study by Kowalczuk et al. (44) excessive workload proved to increase fatigue symptoms and the probability of more recurrent absenteeism.

With regard to the three predictive models proposed, it is observed that the perceived health and vigor subscale variables of the UWES test are predictive, as was the case in other studies (45), and authors pay particular attention to how nurses with a mediocre or poor perception of their health have 4.448 (95% CI = [2.134; 9.273]) times higher risk of psychological distress in the case of PC nurses than those professionals with an optimal perception of health.

Likewise, as in previous studies (46), it is striking how nurses who feel vigor, dedication and absorption less than once a week have 4.428, 4.067, and 3.060, respectively, more risk of presenting distress than healthcare professionals who manifest such variables at least once a week when they have already been working for a while, as is the case. But these same authors agree that, in the short term, high levels of work engagement can negatively affect the health of nurses (46).

Among the limitations that the study may pose are those derived from the methodology used itself. First, there could be a possible selection bias in the study population, as it is subjected to the degree of interest of professionals in participating, in addition to the use of self-administered questionnaires. Researchers should rely on the veracity of the data proposed by the people who have participated in the study. In addition, the type of sampling used, being non-probabilistic, allows to have an orientation of the results but not a representativeness of the sample. In this sense, it should be noted that the results point to associations, but do not allow to establish cause-and-effect relationships as it is a cross-sectional study. Finally, although the objective of the study focuses on nurses, it may be interesting to include other professionals in this environment, including non-health workers, in future research.

## Data Availability Statement

The original contributions presented in the study are included in the article/Supplementary Material, further inquiries can be directed to the corresponding author/s.

## Ethics Statement

The studies involving human participants were reviewed and approved by Ethics Committee of the General Council of Nursing. The patients/participants provided their written informed consent to participate in this study.

## Author Contributions

JG-I, JG-S, MO-M, and YN-A: conceptualization, formal analysis, and investigation. JG-I, MO-M, and YN-A: data curation, resources, and writing—original draft. JG-I, JG-S, and YN-A: methodology and writing—review and editing. JG-I and JG-S: project administration. MO-M: software. JG-S and YN-A: supervision. JG-S and MO-M: validation. JG-I, JG-S, and MO-M: visualization. All authors contributed to the article and approved the submitted version.

## Conflict of Interest

The authors declare that the research was conducted in the absence of any commercial or financial relationships that could be construed as a potential conflict of interest.
