# Supplementary material for: Relationship Between Work Engagement, Psychosocial Risks, and Mental Health Among Spanish Nurses: A Cross-Sectional Study
Source: Front Public Health. 2021 Jan 26;8:627472. doi: 10.3389/fpubh.2020.627472 (PMC7870998; doi:10.3389/fpubh.2020.627472)
Supplement: Supplementary file 1 [file Table_1.DOCX]

Supplementary Material 1

**Supplementary Material 1**. Scoring of the six scale categories: Vigour, Dedication, Absorption, and total, distinguishing patient care groups. Descriptive results of work engagement by dimensions.

|  |  | **1**  **(Sometimes per year)** | | **2**  **(Once or less per month)** | | **3**  **(Sometimes per month)** | | **4**  **(Once a week)** | | **5**  **(Sometimes per week)** | | **6**  **(Every day)** | | **Overall total** | |
| --- | --- | --- | --- | --- | --- | --- | --- | --- | --- | --- | --- | --- | --- | --- | --- |
|  |  | **n** | **%** | **n** | **%** | **n** | **%** | **n** | **%** | **n** | **%** | **n** | **%** | **n** | **%** |
| **Vigour** | PC nurses | 5 | 1.62% | 16 | 5.19% | 14 | 4.55% | 37 | 12.01% | 89 | 28.90% | 147 | 47.73% | 308 | 100% |
|  | EC nurses | 1 | 0.67% | 10 | 6.71% | 23 | 15.44% | 29 | 19.46% | 33 | 22.15% | 53 | 35.57% | 149 | 100% |
|  | Other areas | 21 | 1.68% | 84 | 6.74% | 157 | 12.59% | 256 | 20.53% | 326 | 26.14% | 403 | 32.32% | 1247 | 100% |
| **Dedication** | PC nurses | 6 | 1.95% | 10 | 3.25% | 13 | 4.22% | 25 | 8.12% | 73 | 23.70% | 181 | 58.77% | 308 | 100% |
|  | EC nurses | 3 | 2.01% | 10 | 6.71% | 11 | 7.38% | 19 | 12.75% | 30 | 20.13% | 76 | 51.01% | 149 | 100% |
|  | Other areas | 31 | 2.49% | 64 | 5.13% | 109 | 8.74% | 175 | 14.03% | 322 | 25.82% | 546 | 43.79% | 1247 | 100% |
| **Absorption** | PC nurses | 7 | 2.27% | 6 | 1.95% | 16 | 5.19% | 33 | 10.71% | 75 | 24.35% | 171 | 55.52% | 308 | 100% |
|  | EC nurses | 9 | 6.04% | 6 | 4.03% | 18 | 12.08% | 23 | 15.44% | 33 | 22.15% | 60 | 40.27% | 149 | 100% |
|  | Other areas | 31 | 2.49% | 75 | 6.01% | 97 | 7.78% | 186 | 14.92% | 346 | 27.75% | 512 | 41.06% | 1247 | 100% |
| **Total** | PC nurses | 5 | 1.62% | 10 | 3.25% | 20 | 6.49% | 35 | 11.36% | 83 | 26.95% | 155 | 50.32% | 308 | 100% |
|  | EC nurses | 4 | 2.68% | 10 | 6.71% | 16 | 10.74% | 26 | 17.45% | 38 | 25.50% | 55 | 36.91% | 149 | 100% |
|  | Other areas | 21 | 1.68% | 62 | 4.97% | 158 | 12.67% | 221 | 17.72% | 369 | 29.59% | 416 | 33.36% | 1247 | 100% |

^PC: Primary Care; EC: Emergency Care.^
