# Supplementary material for: Relationship Between Work Engagement, Psychosocial Risks, and Mental Health Among Spanish Nurses: A Cross-Sectional Study
Source: Front Public Health. 2021 Jan 26;8:627472. doi: 10.3389/fpubh.2020.627472 (PMC7870998; doi:10.3389/fpubh.2020.627472)
Supplement: Supplementary file 2 [file Table_2.DOCX]

Supplementary Material 2

**Supplementary Material 2**. Descriptive of the GHQ-12 test for PC, EC, Other areas, and Total of professionals’ services.

| **GHQ-12 (Dichotomous scale)** | **PC nurses** | **EC nurses** | **Other areas** | **Total** |
| --- | --- | --- | --- | --- |
| No of cases | 308 | 149 | 1247 | 1704 |
| Percentage | 18.08% | 8.74% | 73.18% | 100% |
| Mean | 2.94 | 4.17 | 3.87 | 3.73 |
| Median | 2 | 3 | 3 | 3 |
| Mode | 0 | 0 | 0 | 0 |
| Standard deviation | 3.41 | 3.79 | 3.70 | 3.67 |
| Asymmetry coefficient | 1.28 | 0.70 | 0.72 | 0.81 |
| Minimum | 0 | 0 | 0 | 0 |
| Maximum | 12 | 12 | 12 | 12 |
| No of higher atypical | 32 | 0 | 0 | 324 |
| Higher atypical percentage | 10.39% | 0.00% | 0.00% | 19.01% |
| Cases with scores >3 | 94 | 71 | 536 | 701 |
| Scores percentages >3 | 30.52% | 47.65% | 42.98% | 41.14% |

^PC: Primary Care; EC: Emergency Care.^
